# Epidemiological and clinical aspects of bipolar disorders: controversies or a common need to redefine the aims and methodological aspects of surveys

**DOI:** 10.1186/1745-0179-1-4

**Published:** 2005-04-28

**Authors:** Mauro Giovanni Carta, Jules Angst

**Affiliations:** 1 Division of Psychiatry, Department of Public Health, University of Cagliari, Italy; 2 Zurich University Psychiatric Hospital, Zurich, Switzerland

**Keywords:** epidemiology, bipolar disorder, psychiatry, mood disorders, community surveys, prevalence, structured interviews

## Abstract

Data from surveys of large samples showed the lifetime prevalence rates of bipolar disorder around 1.5%. A main question is whether the low prevalence rates of bipolar disorders are not an artefact of the over-diagnosis of depression and under-diagnosis of bipolar-II.

Analysis of the clinician's logical inferential diagnostic process, confirms that the patient does not represent the sole source of useful information because many patients do not experience hypomania as distress but rather as recovery from depression or as a period during which they felt truly well.

Epidemiological data are derived from interviews carried out by lay staff which only reflect the patient's point of view.

The clinical monitoring study carried out alongside the ESEMED project found for the diagnosis of mood disorders, a Kappa agreement (versus clinical interview) which ranged from 0.23 in Spain to 0.49 in France.

If we consider exactly what a Kappa of 0.4 implies for a disorder with an "identified" prevalence rate of 2%, we discover that the prevalence rate may have been under-diagnosed approximately 1.5-fold, so 67% of cases may not have been identified and 50% of the identified cases may be false positives.

It is legitimate to surmise that the prevalence reported by recent (extremely costly) epidemiological surveys may be doubtful.

Which direction should epidemiology take in dealing with the serious matter of bipolar disorders?

Recently, some community surveys were carried out in the USA using the Mood Disorder Questionnaire. In the ensuing debate, one side claimed that the instrument was scarcely accurate when used in the general population, gave rise to numerous false positives and that the high prevalence reported was therefore a mere artefact. The other side defended the results reported by the research studies, on the basis that "positive" cases were homogeneous with regard to the high level of subjective distress, low social functioning and employment and with the high recourse to health care structures.

It is quite probable that the problem lies at the root of the matter, in the definition of the gold standard.

In the present state of our knowledge on course and response to treatment, the current diagnostic thresholds applied for mixed states and hypomanic episodes seem to be unsatisfactory.

It is inconceivable that the diagnostic gold standard should be determined only on the basis of a structured interview of patients alone. But unless there is clinical consensus on the diagnostic threshold for hypomania and mixed states, there can be no consensus on the findings of epidemiological research.

## To our readers

This commentary is the first of a series designed to introduce the new online journal *Clinical Practice and Epidemiology in Mental Health*.

Based on the characteristics that were described in the first editorial and by means of a series of introductory commentaries, the journal will seek to stimulate discussion of the main psychiatric topics facing clinical and epidemiological research across the scientific community. An editorial board of international renown will both chair these debates and actively contribute to this series. .

It is no mere chance that the first commentary deals with bipolar disorders, arguably a leading issue in current psychiatric research. In spite of the importance of the topic, a number of researchers maintain that it is not adequately taken into account in the medical field, probably due to the marked inconsistency in the available epidemiological data.

## Bipolar disorders today

Are we right then in defining the field of bipolar disorders as a poorly recognized medical problem? Apparently not. The World Health Organization has defined bipolar disorders as one of the leading causes of disability throughout the world [1].

In the fields of clinical practice and prevention, it is often underlined that bipolar disorders represent a devastating risk factor for both suicide attempts and suicide itself [2]. It is a well-known fact that subjects affected by this disorder are grossly penalized in the area of employment [3]

## The high costs of bipolar disorders

The financial implications of bipolar disorders are only just beginning to be taken into account.

The first studies carried out on the costs of bipolar disorders indicate expenditures ranging between 24 and 30 billion US$ in the United States over a one year period [4].

Data obtained from an American insurance company reveal how patients affected by bipolar disorders, i.e. 3% of subjects seeking medical assistance, account for 12% of total expenditure [5]. The author of the paper concludes that this is "the most expensive behavioral health care diagnosis". However, the same author reports that this expenditure is largely concerned with the cost of inpatient care of subjects diagnosed years after onset of the disorder, suggesting that the financial burden could therefore be lessened.

On the other hand, very few data are available on indirect costs, although these, too, are estimated to be extremely high. A study performed in the United States in 1991 (published in 1995) reported that indirect costs represented 83% of the total expenditure [6].

## Is the prevalence of bipolar disorder really low?

However, if we analyze data from surveys of large samples at both national and trans-national level, the prevalence rates of bipolar disorder are dramatically low. The ECA study reports a prevalence rate for bipolar disorders of 1.5% in the general population, of which only 0.3% are bipolar II. The National Comorbidity study indicates a lifetime prevalence for mania and hypomania of 1.6% [7]. More recent data from the Netherlands (NEMESIS Study) also suggested a low prevalence rate for bipolar disorder of 1.9% [8]. The as yet unpublished findings reported by the recent multi-center European study ESEMED reveal even lower frequencies, under 1%. Although several reviews of studies performed on smaller samples do not exactly confirm these findings, it is the larger studies which determine the opinions of the managers of health and research programs. Accordingly, bipolar disorders would not appear to have the same degree of impact on public health as major depressive disorders. Indeed, the leading international studies on major depressive disorders show a lifetime prevalence rate ranging from 3 to 17% in western societies, with an upward trend being evidenced in recent research projects [9]. A main question is whether the low prevalence rates of bipolar disorders are not an artefact of the over-diagnosis of depression and under-diagnosis of bipolar-II.

## The definition of bipolar spectrum disorders

The current definitions of mixed state, hypomania and bipolar disorder II are not, however, universally accepted. In clinical practice very few psychiatrists apply a diagnostic threshold for mixed states as high as that indicated by DSM-IV (full criteria for a depressive and manic episode). The impressions held by many clinicians are supported by the findings of a twenty-year longitudinal community study carried out in Zurich [2], which found that depressives with a subthreshold hypomanic syndrome were similar to bipolar II disorders in terms of positive family history for mania, course, comorbidity and treatment rates. Moreover, sub-threshold manic symptoms in adolescence appear to be highly predictive of the subsequent onset of manic episode [10].

## Decisive sources of information and under-diagnosis of bipolar disorders

A further explanation for the apparently low prevalence reported by epidemiological studies may be that the methodological instruments used have led to an under-diagnosis of cases of bipolar disorder.

Analysis of the clinician's logical inferential diagnostic process, particularly when diagnosing bipolar disorder, confirms that the patient does not represent the sole source of useful information. At times, the patient's spouse, a relative or a close friend will refer fundamental information, often because many patients do not experience hypomania as distress but rather as recovery from depression or as a period during which they felt truly well. One of the clinician's tasks is to cross check the statements of the patient and significant others and to act as a mediator between them.

Accordingly, the clinician will attempt to convey relatives' comments to the patient in order to create an awareness that others may view his behavior as pathological. Moreover, in the course of the logical procedure leading to diagnosis, it is not only what the patient says that should be taken into account, but also the way in which he or she says it and the coherence of his or her ideas.

It is not the patient's views that determine the diagnosis, but rather the clinician's judgment. We are all well aware of how far the views of a patient in a hypomanic state may differ from the clinical judgment, particularly in the case of a patient who has never received any form of treatment and who therefore has not yet accepted the medical model of the illness.

In the IOWA STUDY (a prospective investigation of the hereditary nature of schizophrenia and bipolar disorders) the prevalence of mania among relatives of patients affected by bipolar disorders, calculated solely on the basis of diagnostic interviews, was 1.9%; however, when additional sources of information such as clinical records and cross-interviewing of relatives were considered, the rate increased to 5.3% [11].

## Interviews

Epidemiological data are derived from interviews carried out by lay staff, which only reflect the patient's point of view. These instruments may at times be so highly structured that they do not allow any interaction between the clinician's judgment and the diagnostic algorithm. The accuracy of diagnosis of the lay interviews is measured by comparison with the so-called "clinical" interviews, which are again based on the patient as the source of information. Less structured and more clinically-oriented tools such as SCAN [12] are rarely used for the validation of epidemiological interviews.

Basically then, the instruments used to validate epidemiological interviews are semi-structured conversations carried out by clinicians, although the diagnostic algorithm continues to be based to a large degree on patient's response. The gold standard applied for validating the diagnosis of bipolar disorder is much poorer than the free clinical judgment would be.

In view of all these limitations the degree of reliability remains low.

The clinical monitoring study carried out alongside the ESEMED project found for the diagnosis of mood disorders, a Kappa agreement (versus a clinical interview) which ranged from 0.23 in Spain to 0.49 in France [13]. The researchers considered this to be satisfactory!

If we consider exactly what a Kappa of 0.4 implies for a disorder with an "identified" prevalence rate of 2%, we discover that the prevalence rate may have been under-diagnosed approximately 1.5-fold, so 67% of cases may not have been identified and fifty percent of the identified cases may be false positives (table 1).

**Table 1:** Simulation of the agreement between two instruments where Kappa = 0.4 for a condition with a prevalence of 3% (SCID) identified as 2%

	SCID +	SCID -	TOTAL
	Bipolar cases	Not bipolar cases	
CIDI +	1	1	2
CIDI -	2	96	98
TOTAL	3	97	100

It is legitimate to surmise that the prevalence reported by recent (extremely costly) epidemiological surveys may be doubtful.

## The way ahead

Which direction should epidemiology take in dealing with the serious matter of bipolar disorders?

Recently, some community surveys carried out in the USA using the Mood Disorder Questionnaire as a screening tool suggested a prevalence for bipolar disorders of slightly less than 4% [14]. In the ensuing debate, one side claimed that the instrument was scarcely accurate when used in the general population, gave rise to numerous false positives and that the high prevalence reported was therefore a mere artefact [15]. The other side defended the results reported by the research studies, on the basis that "positive" cases were homogeneous with regard to the high level of subjective distress, low social functioning and employment and with the high recourse to health care structures [16]. It is quite probable that the problem lies at the root of the matter, in the definition of the gold standard.

In the present state of our knowledge on course and response to treatment, the current diagnostic thresholds applied for mixed states and hypomanic episodes seem to be unsatisfactory.

If we accept the arguments put forward here, it is inconceivable that the diagnostic gold standard should be determined only on the basis of a structured interview of patients alone. But unless there is clinical consensus on the diagnostic threshold for hypomania and mixed states, there can be no consensus on the findings of epidemiological research.

## Competing interests

The author(s) declare that they have no competing interests.

## Authors' contributions

MGC and JA conceived of the manuscript, and drafted it. Both authors read and approved the final manuscript.
